# Obstacles, research progress, and prospects of oral delivery of bioactive peptides: a comprehensive review

**DOI:** 10.3389/fnut.2024.1496706

**Published:** 2024-11-14

**Authors:** Xinyu Wang, Zeyao Yang, Wangang Zhang, Lujuan Xing, Ruiming Luo, Songmin Cao

**Affiliations:** ^1^School of Food Science and Engineering, Ningxia University, Yinchuan, China; ^2^Key Lab of Meat Processing and Quality Control, MOE, School of Food Science and Technology, Nanjing Agricultural University, Nanjing, China

**Keywords:** bioactive peptides, oral administration, bioavailability, oral delivery systems, peptide transport

## Abstract

Bioactive peptides hold significant potential for enhancing human health, however, their limited oral bioavailability poses a substantial barrier to their widespread use in the food and pharmaceutical industries. This article reviews the key factors influencing the absorption efficiency of oral bioactive peptides, including issues related to bitter taste perception, challenges in gastrointestinal environmental stability, and limitations in transmembrane transport. Furthermore, it highlights the latest technologies, such as osmotic technology, chemical modification, and advanced delivery systems, and discusses their advantages in enhancing the stability of bioactive peptides and facilitating intestinal absorption. In addition, the application and challenges of common delivery systems such as liposomes, emulsions, polymer nanoparticles, and hydrogels in oral bioactive peptide delivery are also discussed. This paper aims to provide a theoretical foundation for scientific research and practical applications of oral delivery of bioactive peptides, thereby promoting the further development of bioactive peptides in the context of human health.

## 1 Introduction

Bioactive peptides are a class of compounds composed of natural amino acids arranged in various combinations, sequences, and spatial conformations. These peptides exhibit diverse physiological activities that are beneficial to the body’s functions. Typically, bioactive peptides range in size from 2 to 20 amino acid residues and have smaller molecular weights compared to proteins, but their bioactivity is often greater than that of proteins (1). Traditional protein digestion theory suggests that proteins can only be absorbed and utilized after being broken down into amino acids upon entering the body (2). However, recent studies have demonstrated that small-molecule peptides are absorbed more readily than proteins. Absorption channels for bioactive peptides exist in the small intestine, allowing these peptides to be directly absorbed and utilized by the body, with an absorption rate that surpasses that of proteins and amino acids. The bioactivity of bioactive peptides is reflected in various aspects, exhibiting regulatory functions such as antihypertensive, antihyperlipidemic, antihyperglycemic, anti-cholesterol, antiviral, and anticancer effects (3).

Although bioactive peptides have the potential to become functional foods and even drugs, their low bioavailability and low activity caused by oral administration are an urgent problem to be solved. The biological activity of a bioactive peptide depends largely on its chemical structure, including amino acid composition, molecular weight, amino acid sequence, and peptide spatial conformation (4). Oral administration of bioactive peptides need to overcome multiple barriers (such as complex enzymatic decomposition in the gastrointestinal tract, changes in pH, adsorption of small intestinal mucus, obstruction of small intestinal mucosal cells, etc.) before they can be absorbed and utilized by the human body. These barriers may cause changes in the sequence and spatial structure of bioactive peptides, resulting in the loss of biological activity of bioactive peptides. Furthermore, these barriers can hinder the absorption and utilization of bioactive peptides, significantly decreasing the amount that enters systemic circulation and performs biological functions in targeted areas.

Currently, various strategies have been developed to enhance the bioavailability of bioactive peptides in the human body. These strategies include chemical structure modifications, permeation enhancers, and colloidal delivery systems, such as liposomes, emulsions, biopolymer nanoparticles, and hydrogels (132). Each of these approaches has its own advantages and disadvantages. For instance, chemical modifications can significantly improve the stability of bioactive peptides; however, they may alter the original chemical structure of the peptides, potentially affecting their biological activity and even leading to the production of harmful substances (5). Although intestinal permeation enhancers (PEs) show good absorption-promoting effects, excessive use can compromise the integrity of the intestinal barrier, and the stability of permeation enhancers in the gastrointestinal tract also requires careful consideration by researchers (6). Encapsulating bioactive peptides using colloidal delivery systems is considered the most promising approach, as it can mask bitterness and overcome many challenges encountered during oral administration, but there are still some problems such as low encapsulation efficiency, poor stability, and poor targeting (7).

In summary, improving the bioavailability of orally delivered bioactive peptides requires a thorough analysis of the advantages and limitations of current delivery strategies. Unfortunately, to date, there remains a lack of systematic collation and comprehensive reviews addressing these issues in the relevant literature. Therefore, this review comprehensively examines the challenges associated with the oral delivery of bioactive peptides, introduces the advantages and disadvantages of existing oral delivery systems, and summarizes the future development trends of these systems. The aim of this review is to provide a valuable reference for subsequent studies on bioactive peptide delivery systems through this in-depth analysis.

## 2 Obstacles to oral administration of bioactive

The oral delivery of bioactive peptides presents several challenges. First, some bioactive peptides may possess a pronounced bitter taste, which can significantly impact patients’ acceptance of oral administration. Second, the digestive tolerance of bioactive peptides within the gastrointestinal tract poses another major challenge for their oral delivery. The variable pH gradient and the complex digestive enzyme system of the gastrointestinal tract can severely affect both the structural integrity and the functional stability of bioactive peptides. Additionally, the intricate defense system formed by the mucus layer, epithelial cells, and microbial community in the gastrointestinal tract is a critical factor limiting the oral bioavailability of these peptides. Furthermore, the unique physicochemical and structural properties of bioactive peptides can also significantly influence their efficacy in oral delivery.

### 2.1 Bitter taste barrier

Bioactive peptides from natural sources are very limited, so most bioactive peptides are produced by enzymatic hydrolysis of proteins. However, proteolysis can not only produce biologically active peptides, but also produce some peptides with a pronounced bitter taste. Generally speaking, bitter taste in food products is not accepted by consumers. The bitterness produced by the hydrolysis process limits the application of active peptides in the food industry, so how to reduce the bitterness is an extremely important issue. The bitter taste of peptides is related to hydrophobic amino acids (8) and their relative molecular masses (9). As early as 1997, Kuhfeld et al. (10) extracted peptides with molecular weights less than 4,000 Da from dried sausages, graded the extracts for sensory evaluation, and found that the higher the intensity of bitterness, the higher the concentration of hydrophobic amino acids in the extracts. Henriksen et al. (11) extracted bitter peptides from commercially available soy protein hydrolysates. The analysis showed that the bitterness of soy peptides was mainly associated with the presence of medium molecular weight peptides in the range of 1,000–4,000 Da, and the bitterness of peptide fractions less than 1,000 Da was lower than that of high molecular weight fractions.

Since the middle of the 20th century, the research on the removal of the bitterness of short protein peptides has gradually increased, and the most common method is masking. Fan et al. (12) used a variety of masking agents for removing bitterness from soy protein hydrolysates, among which xylitol, sucrose, and α-maltodextrin had significant debittering effects. In addition, bitterness can also be removed by destroying the structure of bitter peptides by enzymatic hydrolysis (13), which is widely used in industry because of its high efficiency and no loss of nitrogen. Saha et al. (14) used aminopeptidase to hydrolyze soybean protein isolate with a bitterness value of 3.6 to reduce its bitterness value to 0.4 reducing its bitterness value to 0.4. It is worth noting that the plastein reaction, the reaction in which protease promotes the formation of a gel-like substance from high-concentration protein hydrolyzate under suitable conditions, is an effective debittering method (15, 16). Peptide condensation during plastein reactions can help reduce the bitterness intensity of polypeptides. However, the plastein reaction is not yet applied in industry and needs further exploration.

### 2.2 Barriers of orally administered bioactive peptides in the gastrointestinal tract

#### 2.2.1 Biochemical barrier

**Figure 1:**
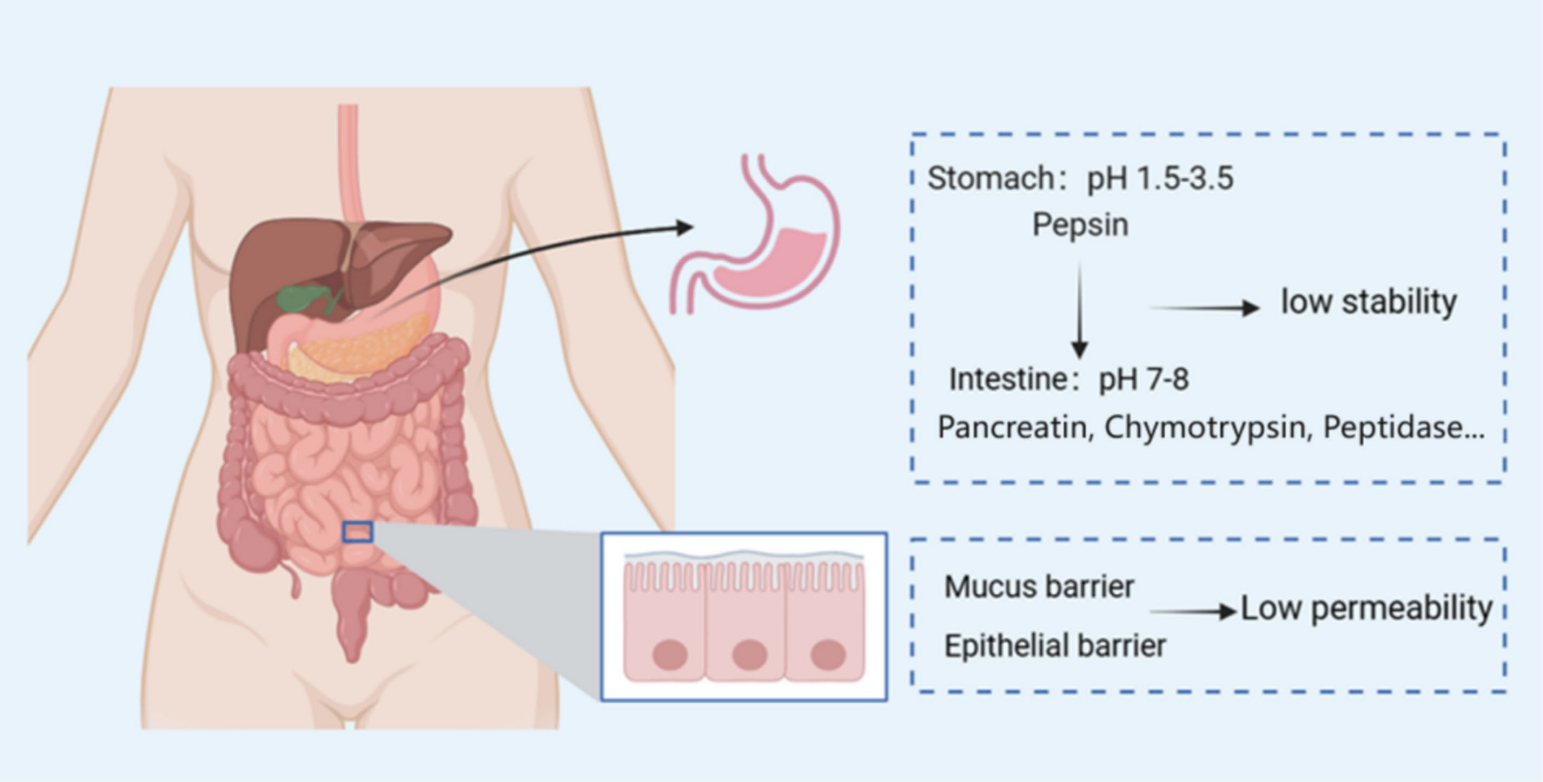
Gastrointestinal disorders affecting peptide absorption.

Two major types of biochemical barriers exist for orally administered peptides: variable pH and gastrointestinal proteases (Figure 1). Orally administered bioactive peptides travel through the oral cavity to the stomach, then to the duodenum, jejunum, ileum, and finally to the colon and rectum (17). Although digestion begins in the oral cavity, due to the extremely short oral action time, the oral cavity not typically cited as a major factor hindering the absorption and utilization of orally administered bioactive peptides. The main factors affecting the absorption and utilization of oral bioactive peptides mainly come from the stomach and small intestine. The first thing to overcome when taking bioactive peptides orally is the variable pH of the gastrointestinal tract. The pH value of gastric juice is 1.5–3.5, that of the duodenum is about 5–6, and that of the jejunum and terminal ileum rises to 7–8 (18). Variable pH gradients have a great impact on the physiological efficacy of some bioactive peptides. The antioxidant activity of the pentapeptide ATSHH from whitefish protein will show a significant decrease trend under acidic conditions (pH = 2) (19).

In addition, after the bioactive peptides reach the stomach, they will stimulate the gastric mucosa to secrete pepsin from the gastric lining cells. Pepsin can hydrolyze the polypeptide with aromatic residues such as phenylalanine, tryptophan, and tyrosine. Bioactive peptides hydrolyzed by pepsin will lose their inherent biological activity. After the bioactive peptide enters the small intestine through the stomach, the trypsin and chymotrypsin present in the small intestine will also specifically hydrolyze the peptide chain (20). The hydrolysis of the above enzymes will change the structure and activity of the bioactive peptide. Li et al. (21) performed *in vitro* simulated digestion experiments on rice protein hydrolyzate and found that the anti-hypertensive IC_50_ (half maximal inhibitory concentration) value of rice protein increased from 140 to 180 μg/mL in the presence of digestive enzymes (pepsin and pancreatic enzymes), indicating that the anti-hypertensive activity of rice protein hydrolyzate was significantly reduced. In addition, after the bioactive peptides reach the stomach, they will stimulate the gastric mucosa to secrete pepsin from the gastric lining cells. Pepsin can hydrolyze the polypeptide with aromatic residues such as phenylalanine, tryptophan, and tyrosine. Bioactive peptides hydrolyzed by pepsin will lose their inherent biological activity. After the bioactive peptide enters the small intestine through the stomach, the trypsin and chymotrypsin present in the small intestine will also specifically hydrolyze the peptide chain (20). The hydrolysis of the above enzymes will change the structure and activity of the bioactive peptide. Li et al. (21) performed *in vitro* simulated digestion experiments on rice protein hydrolyzate and found that the anti-hypertensive IC_50_ (half maximal inhibitory concentration) value of rice protein increased from 140 to 180 μg/mL in the presence of digestive enzymes (pepsin and pancreatic enzymes), indicating that the anti-hypertensive activity of rice protein hydrolyzate was significantly reduced.

#### 2.2.2 Mucus and epithelial barrier

After bioactive peptides are digested in the stomach and successfully reach the small intestine, the intestinal mucus layer covering the intestinal surface is one of the main factors limiting the bioavailability of oral bioactive peptides. The intestinal mucus layer is a kind of intelligent hydrogel with high viscoelasticity and adhesiveness, which contains highly branched polysaccharides and negatively charged mucin (22). The intestinal mucus layer plays a protective role by forming a sieve-like structure on itself. This structure can effectively prevent 10–200 nm particles from passing through the mesh, and has the function of selectively transmitting nutrients (23). Mucin, glycolipids, and glycoproteins in the mucus layer act as both barriers and transmit signals (24). When bioactive peptides reach the intestinal mucus layer, their further diffusion may be affected by mucin adhesion.

After bioactive peptides pass through the mucus layer and reach the surface of epithelial cells, the epithelial cells located under the mucus are another major factor limiting the bioavailability of oral bioactive peptides. The small intestine epithelial cells are a continuous monolayer that separates the intestinal lumen from the underlying lamina propria. There is a tight junction (TJ) between adjacent epithelial cells, which only allows small molecules such as water and ions to pass through. In addition, the small intestine cell membrane acts as a barrier to prevent extracellular substances from freely entering and exiting the cells by selectively absorbing nutrients (25). Based on the above reasons, the small intestinal epithelium is impermeable. Bioactive peptides need to pass through the TJ or intestinal epithelial cell membrane to reach the bloodstream and ultimately bind to the target to exert physiological activity. However, most bioactive peptides cannot effectively penetrate intestinal epithelial cells due to the lack of targeted carrier proteins on the intestinal epithelial cell membrane, which seriously affects the bioavailability of bioactive peptides.

### 2.3 Physical and chemical properties of peptides

The physicochemical properties of peptides are one of the important factors affecting the bioavailability of orally delivered active peptides. The molecular weight and structural characteristics of the peptides can affect their absorption. Compared with short-chain peptides with smaller molecular weights, long-chain peptides are more sensitive to gastrointestinal proteases, which results in long-chain peptides being more easily degraded and absorbed by gastrointestinal digestive enzymes (26, 131). Research by Chen and Li (27) showed that the stability of casein-derived peptides with different molecular weights varies in simulated gastrointestinal tracts. Peptides with a molecular weight greater than 3 kDa are more likely to be degraded during gastric digestion than peptides with molecular weights less than 3 kDa (27). In addition, studies have shown that some short peptides with smaller molecular weights can be transported across intestinal cells through peptide transporters expressed in the intestine, while oligopeptides can be passively transported and absorbed into the body through hydrophobic regions or tight junctions of membrane epithelial cells (28, 130). However, long-chain peptides typically need to be absorbed through endocytosis. Therefore, short-chain peptides are more easily absorbed and utilized by the body.

In addition, the structural characteristics of peptides also play a crucial role in the stability of oral bioactive peptides. The amino acids sequence and structure of bioactive peptides can affect the stability of peptides during digestion, thereby affecting their bioavailability. Savoie et al. (29) found that high levels of proline and glutamic acid in peptide sequences can enhance the resistance of peptides to pepsin and trypsin. Udenigwe (30) research showed that bioactive peptides with a higher β-sheet structure ratio are more sensitive to heat treatment. In addition, the charge of the peptide has been shown to affect the transport of peptides. For example, peptides with neutral amino acid residues can be preferentially recognized by oligopeptide transporter 1 (PepT1) (31). PepT1 is a transporter protein present on the brush like border membrane of the small intestine epithelium. The research of Wang and Li (32) showed that in addition to PepT1 mediated transport pathway, bioactive peptides can also cross small intestinal epithelial cells through endocytic transport and paracellular transport. For example, positively charged hydrophobic antioxidant casein peptides can be transported via endocytosis, whereas negatively charged hydrophilic peptides need to be transported via paracellular pathways.

### 2.4 Absorption mechanism of peptides

**Figure 2:**
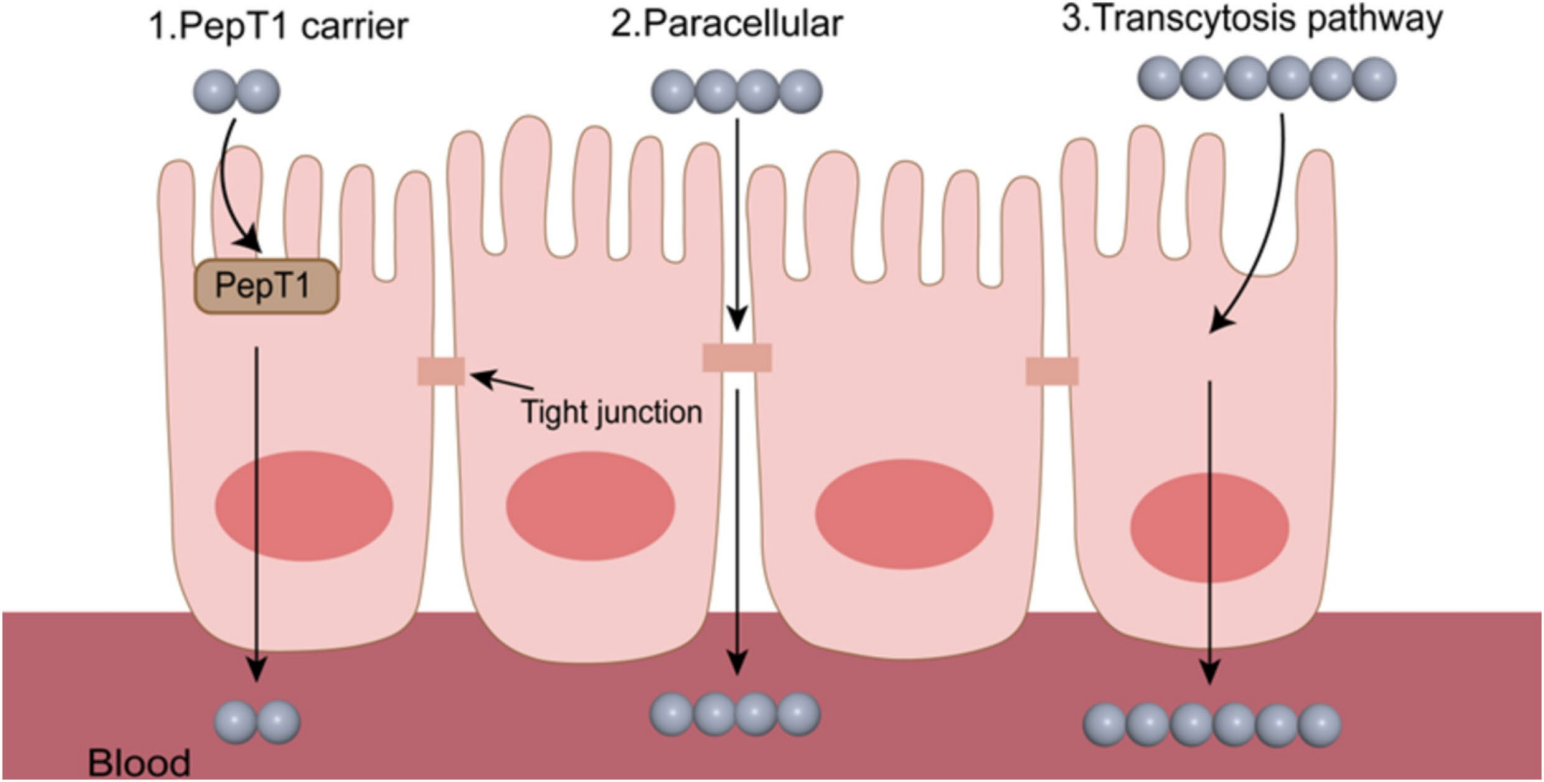
Several transmembrane transport pathways of bioactive peptides.

After successfully overcoming multiple obstacles such as the variable pH environment of the gastrointestinal tract, enzymatic hydrolysis by gastrointestinal digestive enzymes, and adhesion/pre-cleavage of the intestinal mucus layer, bioactive peptides still need to overcome the obstruction of the small intestinal epithelial cells to enter the blood circulation system, which is the prerequisite for the physiological functions of bioactive peptides *in vivo*. There are three main modes of transmembrane transport of bioactive peptides (Figure 2): vector transport, cell bypass transport, and endocytosis transport (33).

#### 2.4.1 Carrier-mediated transport pathway

The carrier-mediated transport pathway primarily relies on oligopeptide transporters (34). The important feature of transporters is that they can select peptides. Transporters have been found to recognize and transport over 8,000 different peptides (35). There are two main types of transporters: PepT1 and PepT2. Both PepT1 and PepT2 can be used for the transport of dipeptides and tripeptides (36). Currently, there are more studies on PepT1 than PepT2 on the transport of polypeptides. PepT1 is mainly expressed in intestinal epithelial cells and is responsible for the transport and absorption of bioactive peptides. As mentioned in the section on the physicochemical properties of peptides, the charge of peptides affects the mode of transport, and PepT1 preferentially recognizes peptides with neutral charge and high hydrophobicity, and preferentially binds residues rich in non-polar amino acids. Fan et al. (37) studied the transport modes of IW, IWH, and IWHHT peptides in Caco-2 cells, which further verified that PepT1 preferred to select small peptides with high hydrophobicity. Table 1 summarizes the transport pathways of different bioactive peptides through the Caco-2 cell model, aiming to provide a solid experimental basis for subsequent research and product development.

**Table 1:** Transport pathway of bioactive peptides through Caco-2 cells.

Bioactive peptides	Function	Source	Transport pathways	Ref
IRW	Anti-hypertensive, Anti-oxidant	Ovotransferrin	PepT1, TJs	(111)
IPP, LKP	Anti-hypertensive	Bovine milk β-casein	PepT1, TJs	(112)
VPP	Anti-hypertensive	Fermented milk	TJs	(113)
IQW	Anti-hypertensive	Ovotransferrin	PepT1, TJs	(114)
LSW	Anti-hypertensive, Anti-inflammatory	Soybean protein	PepT1, TJs	(115)
YPI	Anti-hypertensive	Whey protein	PepT1	(116)
IW	Anti-hypertensive	Myogenic fibers of hens	PepT1	(37)
IWH	Anti-hypertensive	Myogenic fibers of hens	PepT1, TJs	(37)
IWHHT	Anti-hypertensive	Myogenic fibers of hens	TJs	(37)
RVPSL	Anti-hypertensive	Ovotransferrin	TJs	(117)
VLPVP	Anti-hypertensive	Genetic engineering isolation	TJs	(118)
HLPLP	Anti-hypertensive	β-casein	TJs	(119)
VY	Anti-hyperglycemic	Black bean sauce	PepT1, TJs	(120)
VPLVM	Anti-hyperglycemic	Broccoli	PepT1, TJs	(121)
LPEW	Anti-hypertensive	Fermented milk	Transcytosis	(122)
GLLLPH	Anti-oxidant	Corn Gluten	TJs, Transcytosis	(123)
YFCLT	Anti-oxidant	Corn Gluten	TJs, Transcytosis	(123)
LAPSLPKPKPD	Anti-hypertensive	Egg yolk protein	Transcytosis	(124)
β-casein 193–209	Immunomodulatory	Bovine milk β-casein	Transcytosis	(45)
YWDHNNPQIR	Anti-oxidant	Canola protein	Transcytosis	(46)

#### 2.4.2 Paracellular transport pathway

The paracellular transport pathway is currently the most reported passive absorption pathway for bioactive peptides with more than tripeptides (38). The driving force for oligopeptide transport comes from the electrochemical gradient formed by protons as high-energy electrons are transferred along the respiratory chain, and the diffusion process does not require a carrier or energy consumption (39). The paracellular transport pathway is mediated through the TJ between epithelial cells, a tight biological barrier with selective permeability (40). It has been shown that TJ tends to transport negatively charged peptides and is selective for positively charged peptides (41), and bioactive peptides with small hydrophilic molecular weights are more inclined to this transport mode (42). In general, when the molecular diameter of a bioactive peptide exceeds 15 Å, the peptide cannot undergo paracellular transported. However, it is still possible for bioactive peptides with larger molecular sizes to diffuse through TJ if their structures have high conformational flexibility (43). Chiasma has successfully developed an oral formulation of octreotide, named Mycapssa®, utilizing its innovative Transient Permeation Enhancer (TPE™) technology. In this approach, sodium caprate serves as an osmotic enhancer, inducing the reversible opening of tight junctions between intestinal epithelial cells to facilitate the paracellular transport of peptides. The successful development of Mycapssa® not only strongly confirms the feasibility of the paracellular transport strategy for the oral delivery of peptide drugs but also paves the way for further research into the oral delivery of bioactive peptides (44).

#### 2.4.3 Endocytic transport pathway

Endocytic transport is an energy-dependent transcellular transport pathway and is the main transport pathway for long-chain peptides. In this pathway, bioactive peptides are transported into cells through the formation of vesicles formed by invagination of the cell membrane (45). Bioactive peptides with smaller molecules can enter the blood circulation through carrier transport and paracellular pathways, while most large molecule peptides need to be transported through endocytosis. The study by Xu et al. (46) showed that 17-peptide (casein 193–209) can be completely absorbed by the Caco-2 cell monolayer model, and its absorption process is mainly carried out through endocytosis transport. The first step in endocytic transport is the interaction of polypeptides with the cell membranes. Since the cell membrane is composed of a lipid bilayer, endocytic transport is considered an ideal pathway for the transport of lipophilic peptides. The anti-oxidant peptide YWDHNNPQIR is transported across the Caco-2 cell monolayer via endocytosis, primarily because it is composed of hydrophobic amino acids (47). Xiao et al. have innovatively designed and prepared a hybrid liposome system named mExos@DSPE-Hyd-PMPC. This system significantly improves drug encapsulation efficiency and enhances endocytic transport efficacy by effectively integrating functional liposomes with milk-derived exosomes (mExos). Notably, this hybrid liposome exhibits adaptive surface characteristics, enabling it to intelligently adjust its physicochemical properties based on the pH microenvironment of the intestinal mucosal surface. This adaptability facilitates a more efficient endocytic transport process (48).

Notably, research has demonstrated that the hydrophilicity and charge state of bioactive peptides play a significant role in their transport within the body (49). The charge can influence the interactions of bioactive peptides with cell membranes, transport carriers, and other molecules in the gastrointestinal environment. Table 2 summarizes the relationship between various transport mechanisms and the properties of peptides. However, it is important to emphasize that hydrophilicity and charge state are not the only factors determining the transport pathways of bioactive peptides. The transport pathways are also influenced by several other factors, including molecular weight, peptide structure, hydrophobicity, the gastrointestinal environment, and the selection of transport carriers (133).

**Table 2:** Relationship between different transport modes and peptide properties.

Transport pathways	Characteristics		
	Peptide molecular size	Water affinity	Electric charge
PepT1	Dipeptide or tripeptide	Hydrophobic	Neutral charge
TJs	Short-chain peptides	Hydrophilic	Negative charge
Transcytosis	Long-chain peptides	Hydrophobic	Positive electric charge

## 3 Oral delivery systems for bioactive peptides

As mentioned above, the oral administration of bioactive peptides encounters numerous barriers in the human body, which significantly diminish their bioavailability. Therefore, the development of effective oral delivery systems to enhance the bioavailability of bioactive peptides is imperative. An ideal oral delivery system should ensure that the bioactive peptide maintains its integrity before reaching the site of absorption and promotes targeted release at the desired site of absorption. Currently, several prominent oral delivery technologies have been extensively studied and applied to overcome the barriers associated with bioactive peptides delivery in the human body. These oral delivery technologies include permeation promotion technologies, chemical structural modifications, colloidal delivery systems, etc.

### 3.1 Permeation promotion technology

One of the biggest obstacles to oral administration of bioactive peptides is the poor permeability of intestinal epithelial cells to bioactive peptides. Permeation enhancers (PEs) are substances that can temporarily increase intestinal permeability and promote the penetration of bioactive peptides through the intestinal epithelium (47). Currently, over 250 substances have been investigated in clinical research as PEs for the oral delivery of bioactive peptides, such as surfactants, fatty acids, bile salts, and cell-penetrating peptides (50). Based on their mechanisms of action, PEs are mainly divided into two categories (51). The first category mainly acts on the TJ between epithelial cells and achieves paracellular transport of bioactive peptides by opening the TJ between epithelial cells. The second category is to promote the transmembrane transport of bioactive peptides by increasing the permeability of the cell membrane. Table 3 lists some typical PEs and their respective mechanisms of action. It is worth noting that some specific PEs can act on both pathways at the same time, such as sodium decanoate, bile salts and chitosan. In addition, although PEs are generally considered safe and non-toxic, the additive dosage of PEs still needs to be strictly controlled when using them. Excessive use of PES can cause excessive changes in the permeability of intestinal epithelial cells, which will eventually induce local inflammation or long-term damage to intestinal epithelium (52). For example, calcium chelators can cause Ca^2+^ depletion in the body, thereby damaging actin filaments, altering adherens junctions and reducing cell adhesion (53).

**Table 3:** Typical PEs for three different mechanisms.

Categories	Mechanism	PEs	Ref
1	Opens the paracellular pathway to facilitate transcellular transport	EDTA	(125)
		Citric acid	(125)
2	Increasing cell membrane permeability to facilitate transcellular transport	SNAC	(126)
3	Simultaneous enhancement of both pathways	Bile salts	(127)
		Sodium caprate (C10)	(128)
		Chitosan	(129)

Cell-penetrating peptides (CPPs), as an important branch of penetration enhancers, are mainly polypeptides ranging from 5 to 30 amino acids, which transport bioactive peptides across the membrane by penetrating the cell membrane or endocytosis (54). Currently, researchers have designed or identified more than 100 peptides that can effectively promote the transport of biological macromolecules across cell membranes. In practical applications, nucleotides, bioactive peptides, and other biologically active substances are prone to lose their activity in the systemic circulation. Encapsulating such substances in nanoparticles can greatly enhance their stability *in vivo*. However, the presence of the cell membrane hinders the uptake of bioactive substances by target cells. CPPs provide researchers with a new direction of exploration. Studies have shown that combining CPPs with nanoparticles can further enhance the transcellular delivery of bioactive peptides and effectively improve the uptake of bioactive substances by target cells. Knoll et al. (55) developed a new type of CPP-modified nanostructured lipid-based carrier, and experimental results demonstrated that this new type of coated nanocarrier can improve the uptake of bioactive substances by cells. The *in vivo* toxicity of CPPs is not yet fully understood, but a small number of published animal studies and several CPP formulations approved for clinical trials demonstrate the general safety profile of CPP molecules at study doses (56). Nevertheless, no CPP-encapsulated drugs have entered clinical trials, and further research is needed to evaluate their *in vivo* delivery effects (Table 4).

**Table 4:** Advantages and disadvantages of four delivery systems.

Categories	Advantages	Disadvantages
Liposomes	Adjustable structure	Lack of stability
	Surface modifiable	High production cost
Emulsion	High bioavailability	Structural instability
Polymer nanoparticles	Structural stability	Complex preparation
	Surface modifiable	Potential toxicity
Hydrogel	Biocompatible	Mechanical strength
	Controlled release	

### 3.2 Chemical structural modifications

Bioactive peptides are a type of molecules that are relatively easy to modify in chemical structure. Chemical modification can significantly improve the stability of bioactive peptides. The more commonly used chemical modification methods are PEGylation and cyclization (57). PEGylation is a chemical modification technique that involves the covalent attachment of polyethylene glycol (PEG) molecules to biological macromolecules, such as proteins and peptides. This process aims to optimize the physicochemical properties and biological characteristics of these biomolecules. For bioactive peptides, the incorporation of PEG can significantly enhance their water solubility, thereby improving their solubility in physiological environments, which is essential for effective absorption and distribution. Furthermore, PEG, being an inert polymer, effectively protects peptide drugs from enzymatic degradation, leading to a substantial increase in the retention rate and bioavailability of bioactive peptides. Additionally, the increase in molecular weight resulting from PEGylation reduces the renal clearance rate of peptide drugs, thereby prolonging their half-life in the body and decreasing the frequency of administration (58). Zhou et al. (59) demonstrated that when the HM-3 peptide was modified with methoxy-PEG-aldehyde, its half-time was extended by 5.86 times in male SD rats. Wang (60) similarly showed that after pegylation, the CPU-HM peptide exhibited higher *in vivo* activity and a longer half-time.

Cyclization is another commonly used method for chemical modification of bioactive peptides. By creating a cyclic structure, cyclization eliminates the exposed N- and C-terminals in peptide molecules, rendering them less susceptible to enzymatic degradation (61). Desmopressin is an analog obtained by cyclization of vasopressin, which is more resistant to enzymatic degradation than vasopressin (62). Similarly, cyclized opioids exhibit longer half-life and higher metabolic stability (63). In addition, cyclic structural peptides have better permeability than linear structural peptides. The cyclic structure is more compact than the linear structure, which reduces the collision of the cyclic structure peptide in the solution and ultimately allows it to pass through the epithelial barrier faster (64).

In addition to debittering, the plastein reaction mentioned above also provides a feasible method for the modification of peptides. Studies have shown that plastein reactions can enhance the activity of angiotensin-converting enzyme (ACE) inhibitory peptides. Song et al. (65) used plastein reactions to modify hazelnut peptides, and the results showed that the ACE inhibition rate of the modified products was significantly improved. Similarly, Jiang et al. (66) employed plastein reaction to modify ACE inhibitory peptides derived from sea cucumbers, and found that the modified peptide showed significantly enhanced thermal stability, and the thermal transition temperature of the modified peptide increased from 120°C to 134°C. These studies indicate that plastein reaction is a promising strategy to induce structural modifications to improve the biological activity of peptides. However, the application of plastein reactions in peptide modification is not immature at present, and research on peptide sequence changes after plastein reactions is relatively limited. Regardless, when modifying the chemical structure of bioactive peptides to improve their bioavailability, it is necessary to pay attention that the modification process cannot affect the original functions of the bioactive peptides and to avoid the generation of harmful substances.

### 3.3 Colloidal delivery system

**Figure 3:**
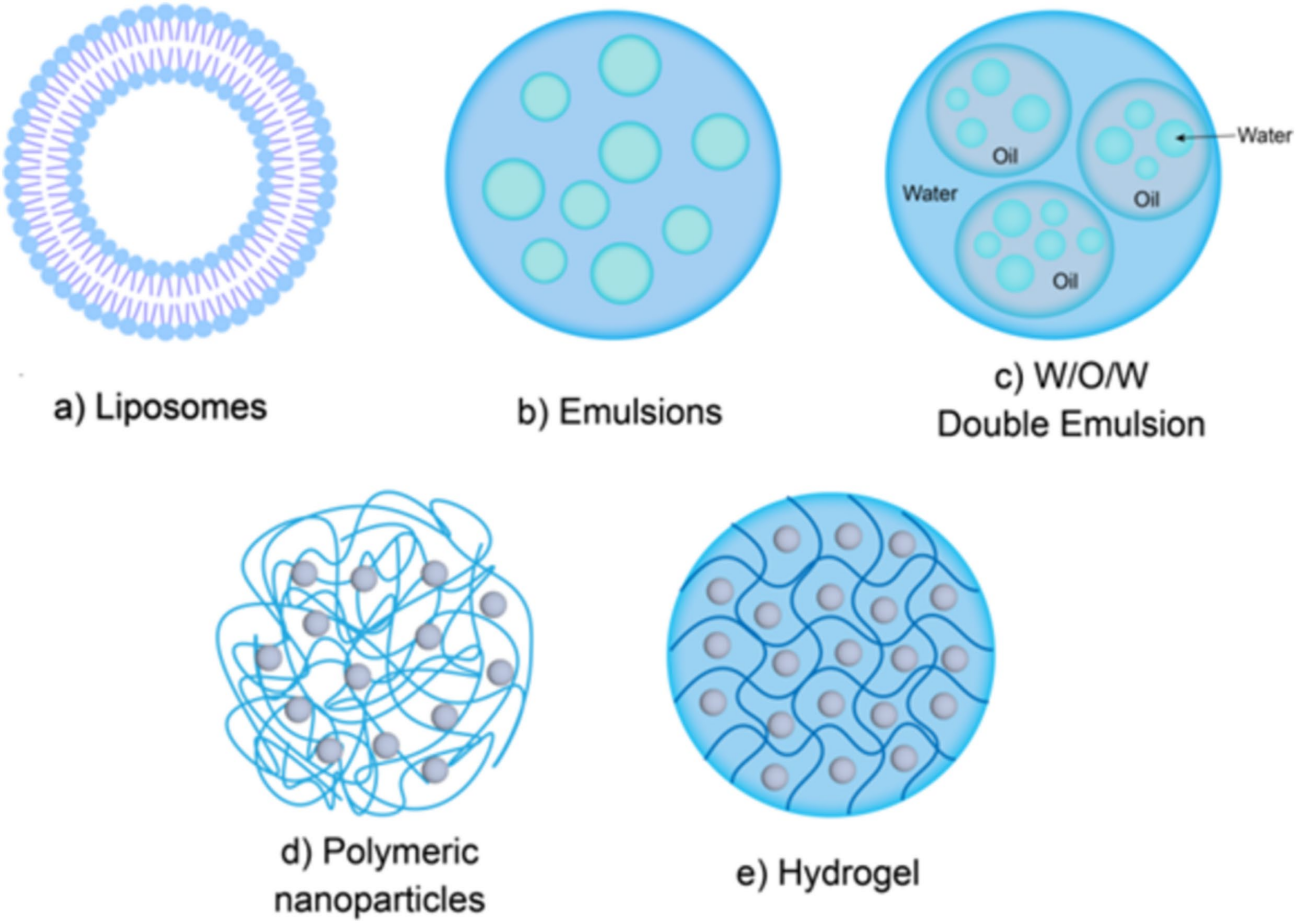
Colloidal delivery system structure. Liposomes **(a)**; Emulsions **(b,c)**; Polymer nanoparticles **(d)**; Hydrogels **(e)**.

Due to the susceptibility of bioactive peptides to loss of physiological activity under different pH values and the action of digestive enzymes in the body, using a delivery system to encapsulate bioactive peptides can effectively eliminate the bitter taste while improving the stability of peptides in systemic circulation. Colloidal delivery systems have been widely applied in the delivery of bioactive peptides. Common colloidal delivery systems include liposomes, emulsions, polymer nanoparticles, and hydrogels, as illustrated in Figure 3.

#### 3.3.1 Liposomes

Liposomes are a kind of spherical closed vesicle formed by concentric phospholipid molecules linked end to end through hydrophobic interactions, which can protect the loaded materials from being broken down by enzymes and improve their bioavailability in the body (Figure 3a) (67). Gong et al. (68) the bioavailability of peanut peptides was effectively improved after being encapsulated in nanoliposomes. The main reason is that the nanoliposomes prepared in this study exhibited good stability under different pH conditions and different morphologies, which allows the peanut peptides encapsulated in the nanoliposomes to retain a relatively complete structure and high ACE inhibitory activity. Compared with other delivery systems, liposomes have the advantages of easy encapsulation, large encapsulation capacity, and minimal residual organic solvents. Liposomes can encapsulate both hydrophobic and hydrophilic bioactive peptides. Hydrophobic peptides can be embedded within the phospholipid bilayer, while hydrophilic peptides can be encapsulated in the aqueous core (69).

**Figure 4:**
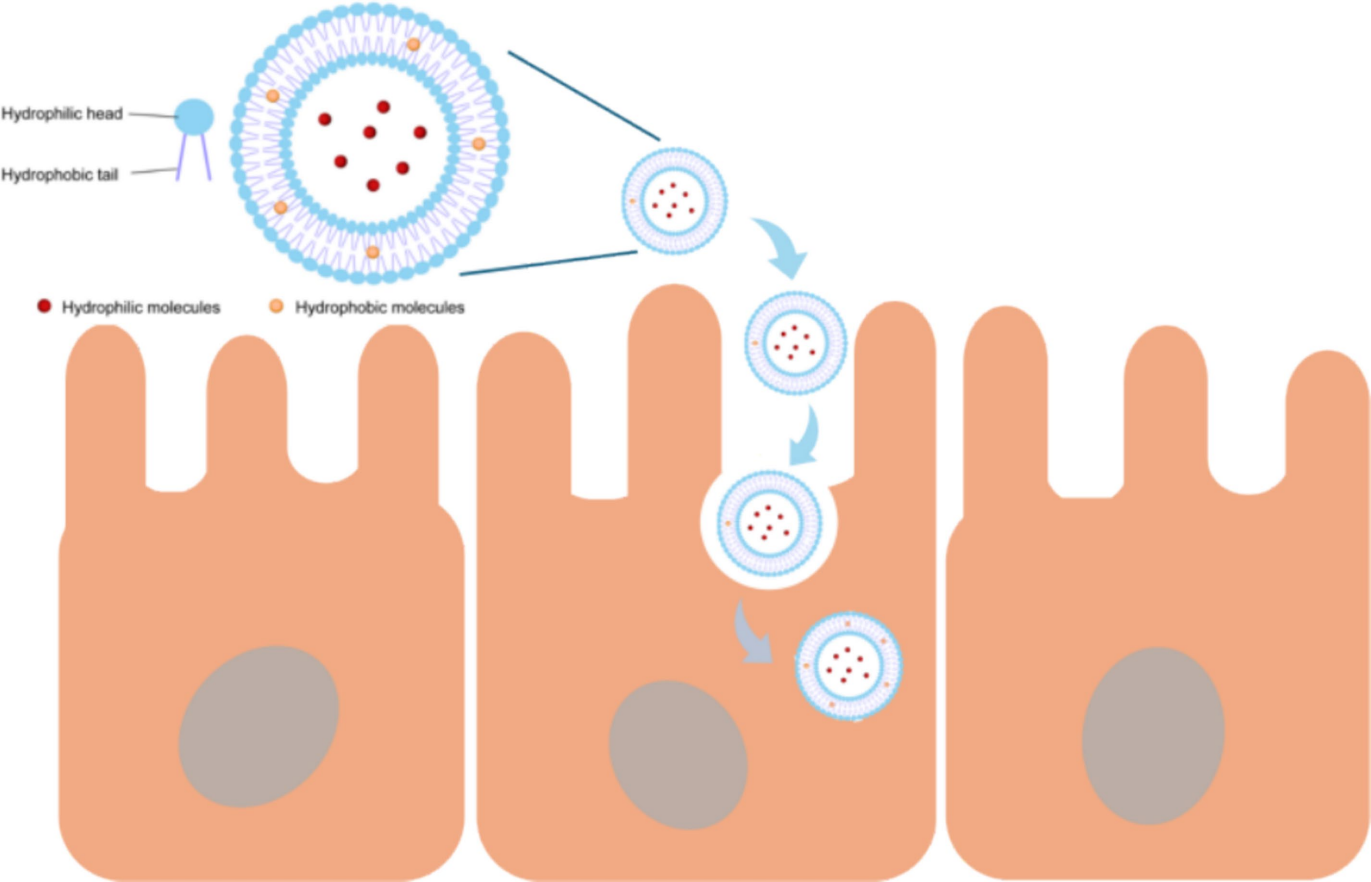
Liposomes deliver substances into cells through vesicle-based delivery.

However, liposomes also have some limitations. Firstly, the phospholipid membrane of liposomes is sensitive to adverse factors such as high temperature, enzymes, and ionic strength. These adverse factors may cause the liposomes to decompose during storage or before reaching the small intestine, causing the bioactive peptides wrapped inside to leak out in advance (70). To overcome this limitation, researchers have found that surface modification of liposomes with polymers such as chitosan, pectin, and polyethylene glycol can effectively improve the stability and sustained release ability of liposomes (71). Ramezanzade et al. (72) developed a novel composite nano-carrier of triphosphorus sodium cross-linked chitosan coated liposomes, and differential scanning calorimetry showed that this composite nano-carrier had better thermal stability than ordinary liposomes. Wu et al. (73) used sodium alginate (SA) to coated liposomes containing DPP-IV inhibitory collagen peptides and found that compared with uncoated liposomes, SA-coated collagen peptide liposomes exhibited higher storage stability, gastrointestinal stability and transcellular permeability. Secondly, due to the large size structure of liposomes, they may not be absorbed by intestinal epithelial cells, and the penetration mechanism of liposomes is not yet clear. Therefor the best approach is to choose vesicles as small as possible for the delivery of active substances, with particle diameters below 100–200 nm (Figure 4) (74). Additionally, cationic charged liposomes are often chosen to deliver bioactive substances because they are more easily attracted to the negatively charged mucus layer. Cuomo et al. (75) employed liposomes for the oral delivery of all-trans-retinoic acid and observed that cationic liposomes could interact with saliva in the oral cavity, which carries a net negative charge. Importantly, when cationic liposomes were coated with mucoproteins from oral saliva, the charge on the cationic surface interaction changed from positive to negative. This prevented the liposomes from being attracted to the negatively charged mucus layer during other stages of digestion, providing further protection for the loaded molecules.

#### 3.3.2 Emulsion

An emulsion is a thermodynamically unstable colloidal dispersion formed by two immiscible liquids (usually oil and water), in which one liquid is dispersed as small droplets in the other liquid (76). According to their structural characteristics, emulsions can be divided into single-layer emulsions (water-in-oil, oil-in-water) (Figure 3b) and multi-layer emulsions (water-in-oil-in-water, oil-in-water-in-oil) (Figure 3c) (77). As a complex multi-phase system, multi-layer emulsion has various system types, among which W1/O/W2 is the most commonly used in food. The main structural state of W1/O/W2 type emulsions is that small water droplets (internal water phase, W1 phase) are trapped in larger oil droplets, and are subsequently dispersed in the external water phase (W2 phase). Multi-layer emulsions are complex multiphase systems. W1/O/W2 type is more common in food, where small water droplets (inner aqueous phase, W1 phase) are trapped in larger oil droplets, which are then dispersed in the outer aqueous phase (W2 phase) (78). Like singlelayer emulsions, the formation of multilayer emulsions also requires the addition of emulsifiers. Previous studies have found that the type of emulsifier can affect the stability of multilayer emulsions. Jo et al. (79) found that the hydrophilic and lipophilic balance value of the emulsifier can significantly affect the stability of W1/O/W2 emulsion loaded with collagen peptides, and emulsifiers with significant amphiphilicity can make W1/O/W2 emulsion more stable. Ying et al. (80) used polyglycerol ricinoleate and modified starch as emulsifiers to successfully prepare an emulsion system with a soybean peptide encapsulation rate of more than 80%. The results of *in vitro* simulated gastrointestinal digestion showed that the emulsion system showed strong resistance to the decomposition of pepsin, and the retention rate of soybean peptide was higher than 70% after simulated gastric digestion. In some cases, even with the addition of emulsifiers, the properties of multilayer emulsions are still not stable enough. This is because the system has two interfaces with a large interfacial area, making the multiphase structure prone to destruction during storage (81). Currently, there are various methods to stabilize the structure of multiple emulsions. One effective method to improve the stability of multiple emulsions is to add proteins or polysaccharides to limit the movement of components. For example, the addition of gelatin to multiple emulsions could significantly improves their stability (82). Furthermore, studies have shown that emulsion delivery systems not only improve the gastrointestinal stability of peptides, but also have the characteristics of masking the bitter taste of bioactive peptides (79). Gao et al. (83) used water-in-oil high internal phase emulsions (W/O HIPE) to encapsulate bitter peptides and found that W/O HIPE had a significant masking effect on the bitter taste of peptides.

Although both single-layer emulsions and multi-layer emulsions need to be stabilized by adding emulsifiers, some synthetic low molecular weight surfactants still need to be considered for their potential harm to the human body (84). Specifically, surfactants with a high HLB (Hydrophilic–Lipophilic Balance) value may disrupt the skin barrier due to their strong interfacial activity, which can increase the skin’s permeability to harmful substances, leading to skin irritation and even triggering allergic reactions and skin inflammation. Secondly, during the preparation of emulsions, although surfactants are renowned for their emulsifying properties, there is also a risk of causing emulsion instability, such as phase separation, coalescence, or creaming. These instability phenomena not only affect the appearance and texture of the product but may also compromise its actual efficacy. Moreover, the interactions between surfactants and bioactive ingredients may lead to structural changes in the bioactive components, resulting in the loss of their original functions, which is crucial for maintaining the integrity of bioactive ingredients. Surfactants may interfere with the permeability and retention time of bioactive components, thereby affecting their distribution and metabolism within the organism, ultimately reducing their bioavailability and therapeutic effects (84). Therefore, researchers have been on the way to seek other safer methods to stabilize the emulsion structure. At this time, a special emulsion, Pickering emulsion, came into the attention of researchers. Cai et al. (85) found that the natural Pickering emulsion system formed by composite nanoparticles that interacted/conjugated antimicrobial peptide Parasin I with chitosan significantly improved the stability and antibacterial activity of Parasin I. The solid particles in Picorling emulsions are irreversibly adsorbed on the surface of the emulsion droplets and play a role in stabilizing the emulsion system. This characteristic of Picorling emulsion avoids the use of surfactants, so its advantage is that there is no need to consider the safety of surfactants in food systems (86). In view of the characteristics and high safety of Pickering emulsions, it has a large application space in the field of bioactive substance delivery, but its specific mechanism of action and application characteristics still require further extensive research.

#### 3.3.3 Polymer nanoparticles

Polymer nanoparticles are solid colloidal particles with an average particle size ranging from 10 to 1,000 nm (Figure 3d). Polymer nanoparticle delivery system is a kind of system that uses natural, semi-synthetic or synthetic polymer nanoparticles as delivery carriers to load bioactive substances through non-covalent methods such as electrostatic adsorption, hydrophobic interaction, hydrogen bonding and so on (87). Compared to lipid-based carriers and emulsions, polymer nanoparticles have a simple preparation process, smaller system size, better stability which can protect bioactive peptides from being decomposed in harsh gastrointestinal environments (88), thereby improving the oral bioavailability of bioactive peptides. Additionally, high lipid intake may in due obesity and cardiovascular diseases (89), while the commonly used materials of polymer nanoparticles are proteins, polysaccharides and their composite derivatives, such as gelatin, sodium alginate, chitosan, and their derivatives, etc. Thus, polymer nanoparticles are more healthier and easilier to be accepted by consumers. Currently, various polymer nanoparticle delivery systems have been designed and applied to bioactive peptides delivery. Zhu et al. (90) used lysozyme-xanthan gum nanoparticles as carriers of selenium-containing peptides and prepared lysozyme-xanthan gum-selenopeptide composite nanoparticles. *In vitro* release test results showed that the composite nanoparticles successfully delayed the release of selenium-containing peptides and improved their *in vitro* antioxidant activity. Uhl et al. (91) developed a surface-modified PLA nanoparticles that can be loaded with liraglutide, which increased the oral bioavailability of liraglutide by 4.5-fold.

Some polymers can reversibly open TJs between intestinal epithelial cells, help bioactive peptides to be transported through the paracellular pathway, and promote the penetration and absorption of bioactive peptides, such as chitosan and its derivatives (92). In addition, chitosan also has good degradability and is one of the commonly used materials for constructing polymer nanoparticle delivery systems (93). Auwal et al. (94) used sodium tripolyphosphate cross-linked chitosan nanoparticles as the carrier to encapsulate ACE-inhibitory peptides, and found that not only the physical and chemical stability of the peptides was significantly improved *in vitro*, but also the ACE inhibitory effect of the peptides was significantly improved after simulated gastrointestinal digestion. Han et al. (95) prepared a pH-sensitive complex through the electrostatic self-assembly of chitosan derivative N-trimethyl chitosan, peanut peptide, and sodium alginate. This complex exhibited a regular spherical shape with good stability, and the highest entrapment efficiency for peanut peptide reached 91%.

#### 3.3.4 Hydrogel

Hydrogel is a highly cross-linked hydrophilic polymer with a three-dimensional network structure and abundant pores that can absorb and retain a large amount of water (96) (Figure 3e). A hydrogel system is a very effective delivery system for bioactive peptides, which can be prepared by mixing bioactive peptides with a solution containing biopolymer molecules before gel formation, or also by loading bioactive peptides into a microgel after microgel formation (97). Ma et al. (98) developed a novel type of fish skin gelatin-based hydrogel that successfully loaded codfish peptides after gel formation and exhibited good mechanical properties and biocompatibility. Because different types of materials have greatly different molecular and physicochemical properties, the physical and chemical differences of materials have a greater impact on the encapsulation effect of the system. Therefore, when preparing hydrogels, materials need to be selected according to specific purposes and applications. Protein and polysaccharide are commonly used materials for the preparation of ingestible food-grade microgels. Huang et al. (99) used the emulsion template method to successfully loaded ACE inhibitory peptides into biopolymer microgels composed of chitosan and alginate, which effectively reduced the *in vitro* release rate of ACE-inhibitory peptides. Ma et al. (100) used hydrogel made of alginate and chitosan to contain sericin with anti-inflammatory activity, and animal experiments showed that sericin loaded by hydrogel could more effectively alleviate ulcerative colitis in mice. These experimental results indicate that hydrogels have great potential in oral delivery systems.

In addition, pH, temperature and other stimuli will lead to the morphological changes of some polymer hydrogels, which will eventually lead to the phase transition of hydrogels (101). The hydrogels with this phenomenon are called smart hydrogels, which can respond to environmental stimuli, also known as environmentally responsive hydrogels. Environmentally responsive hydrogels can make corresponding shrinkage and swelling changes when single or multiple changes occur in external temperature, pH, light, electric field, salinity and other conditions, ultimately achieving targeted release of bioactive peptides (102). The environmental responsiveness of smart hydrogels shows important application potential and value in the field of substance delivery. Specifically, some temperature responsive smart hydrogels can exhibit different morphologies through corresponding phase transitions at elevated or low temperatures depending on the ambient temperature. This temperature responsiveness allows the hydrogel to adjust the position and rate of drug release in response to fluctuations in body temperature or environmental temperature, resulting in precise delivery of internal embedding. For example, Chuang et al. (103) cleverly designed a thermosensitive hydrogel based on the fact that tumor tissue is slightly hotter than normal tissue. This hydrogel will precisely undergo phase transition and release the embedded drug in the high temperature environment of the tumor site, allowing effective tumor treatment with minimal drug damage to normal tissues. In addition, there are some pH-responsive smart hydrogels that can adjust their morphology or properties according to changes in environmental pH, a property that enables the embedded material to respond to release in a specific pH environment, such as the slightly acidic environment of tumor tissue or the acidic environment of the stomach. Xie et al. (104) designed a pH-sensitive hydrogel that expands and releases drugs in the acidic environment of the stomach, which could facilitate precision treatment of gastric ulcer sites. In addition to the temperature and pH response, some smart hydrogels can undergo morphological changes upon the induction of light, which are called photoresponsive hydrogels. In the treatment of skin diseases, Huiwen et al. (105) use photosensitive hydrogels to deliver drugs precisely to lesions, which can significantly reduce the damage of drugs to surrounding normal tissues and improve the accuracy and safety of treatment.

Due to their unique environmental responsiveness, smart hydrogels have the ability to precisely regulate the drug release process, which makes them show broad application prospects in the field of drug delivery. Similarly, with appropriate design and preparation strategies, smart hydrogels are also suitable for quantitative, timed, and site-directed delivery of bioactive peptides. Ye et al. (106) found that the pH-responsive carboxymethyl cellulose/polyvinyl alcohol hydrogel effectively prevented the release of soy peptides in the stomach and could basically achieve the directional release of soy peptides in the intestine. This precise delivery strategy not only enhances the retention rate of bioactive peptides but also significantly improves their bioavailability, thereby optimizing therapeutic effects. In addition, it needs to be acknowledged that although smart hydrogels can effectively control the directional release of bioactive peptides, because the human body environment is complex and changeable, the changes and safety of smart hydrogels in the body need to be further studied.

Another, it needs to be acknowledged that hydrogels also have some disadvantages that are difficult to avoid. Typically, hydrogels are very porous and have weak structural strength, which allows bioactive peptides (especially small peptides) to easily diffuse out of them. At present, some studies have shown that improving the capture rate of bioactive peptides by hydrogels by ensuring that the pores are small enough or enhancing the interaction between bioactive peptides and the biopolymer network within the microgel (107). Two polymers with complementary properties can form a double crosslinked hydrogel to increase the stability of the hydrogel (108). Chen et al. (109) successfully prepared strong gelatin hydrogels by dual-crosslinking gelatin with transglutaminase and carrageenan, which improved the mechanical properties and thermal stability of gelatin hydrogel. In addition, since hydrogels are mostly hydrophilic substances, they have certain limitations when embedding hydrophobic substances. Studies have found that polymerizing hydrogels with nanoparticles, micelles and cyclodextrins can significantly improve the encapsulation rate of hydrophobic substances in hydrogels. Shabkhiz et al. (110) successfully encapsulated a β-cyclodextrin inclusion complex containing glycyrrhizic acid and thyme essential oil into alginate hydrogel beads, increasing the peptide encapsulation rate to 89%. However, there are few reports on the use of this technology in bioactive peptide entrapment, and further investigation is required. In summary, with the further development of smart hydrogel delivery systems, more innovative breakthroughs will be achieved in the application of smart hydrogels in the delivery of bioactive peptides.

## 4 Conclusions and outlook

Bioactive peptides have garnered significant attention from researchers due to their diverse physiological activities. However, the bioavailability of orally delivered bioactive peptides is severely restricted by the natural barriers of the gastrointestinal digestive system, as well as the physical and chemical properties of the peptides themselves. To enhance the stability and bioavailability of oral bioactive peptides within the gastrointestinal environment, various strategies have been explored, including chemical structure modification, the use of penetration enhancers, and colloidal delivery systems (such as liposomes, emulsions, biopolymer nanoparticles, and hydrogels). Nevertheless, each strategy presents distinct limitations in practical applications.

### 4.1 Limitations of delivery strategies

Although chemical modification can effectively enhance the stability of bioactive peptides, alterations in their chemical structure may reduce biological activity or even result in the formation of harmful substances. PEs possess a strong ability to promote absorption; however, inappropriate use can compromise the integrity of the intestinal barrier and significantly impact intestinal health. Liposomes, which mimic the structure of biological membranes, facilitate interactions with cell membranes, thereby offering substantial advantages in improving drug bioavailability and targeting. Nevertheless, liposomes exhibit poor structural stability and are susceptible to external factors that can lead to rupture, fusion, and leakage of their contents. Additionally, the drug loading capacity of liposomes is often suboptimal due to limitations related to molecular size, charge, and hydrophobicity. Emulsions can effectively enhance the solubility and stability of drugs, but they face challenges such as poor dispersion stability and low bioavailability. Polymeric nanoparticles have garnered considerable attention due to their controllable particle size, excellent stability, and biocompatibility. However, improvements are still needed in their drug loading capacity, drug release efficiency, and targeting capabilities. Smart hydrogels exhibit high environmental responsiveness; however, their stability within the digestive system and the controlled release of embedded materials restrict their practical applications.

### 4.2 Future research trends

Recent research indicates that a single delivery system is insufficient to overcome all delivery challenges. As a result, hybrid delivery systems that combine various delivery methods are anticipated to emerge as a major research focus in oral delivery moving forward. With consumers increasingly prioritizing safety and health, the main research emphasis for the oral delivery of bioactive peptides will be on discovering natural, edible, and biocompatible materials that have low toxicity to serve as delivery carriers. Moreover, current design approaches for oral delivery systems mainly concentrate on overcoming the gastrointestinal barrier, while the targeting features of these systems have not been thoroughly investigated. As a result, a key area of research in the oral delivery of bioactive peptides will focus on creating targeted homeostasis within these systems. Additionally, most existing data on the oral delivery of bioactive peptides has come from *in vitro* or animal studies, with a lack of relevant clinical data. To effectively evaluate the impact of oral delivery systems for bioactive peptides on human health, clinical studies are necessary to determine if prolonged use of these systems could result in unexpected side effects *in vivo*. With ongoing technological advancements, it is expected that new hybrid delivery systems will be developed, leading to improved delivery of bioactive peptides.
